# Sound pressure levels generated at risk volume steps of portable listening devices: types of smartphone and genres of music

**DOI:** 10.1186/s12889-018-5399-4

**Published:** 2018-05-01

**Authors:** Gibbeum Kim, Woojae Han

**Affiliations:** 1 Department of Speech Pathology and Audiology, Graduate School, Hallym University, Chuncheon, 24252 Republic of Korea; 2 Division of Speech Pathology and Audiology, Research Institute of Audiology and Speech Pathology, College of Natural Sciences, Hallym University, Hallymdaehakgil 1, Chunchon, 24252 Republic of Korea

**Keywords:** Risk volume level, Personal listening device, Smartphone, Music genre, Noise-induced hearing loss

## Abstract

**Background:**

The present study estimated the sound pressure levels of various music genres at the volume steps that contemporary smartphones deliver, because these levels put the listener at potential risk for hearing loss.

**Methods:**

Using six different smartphones (Galaxy S6, Galaxy Note 3, iPhone 5S, iPhone 6, LG G2, and LG G3), the sound pressure levels for three genres of K-pop music (dance-pop, hip-hop, and pop-ballad) and a Billboard pop chart of assorted genres were measured through an earbud for the first risk volume that was at the risk sign proposed by the smartphones, as well as consecutive higher volumes using a sound level meter and artificial mastoid.

**Results:**

The first risk volume step of the Galaxy S6 and the LG G2, among the six smartphones, had the significantly lowest (84.1 dBA) and highest output levels (92.4 dBA), respectively. As the volume step increased, so did the sound pressure levels. The iPhone 6 was loudest (113.1 dBA) at the maximum volume step. Of the music genres, dance-pop showed the highest output level (91.1 dBA) for all smartphones. Within the frequency range of 20~ 20,000 Hz, the sound pressure level peaked at 2000 Hz for all the smartphones.

**Conclusions:**

The results showed that the sound pressure levels of either the first volume step or the maximum volume step were not the same for the different smartphone models and genres of music, which means that the risk volume sign and its output levels should be unified across the devices for their users. In addition, the risk volume steps proposed by the latest smartphone models are high enough to cause noise-induced hearing loss if their users habitually listen to music at those levels.

## Background

According to a World Health Organization (WHO) report, millions of adolescents and young adults are at risk of developing hearing loss because of their frequent exposure to high sound levels in noisy environments such as nightclubs and discotheques and the unsafe use of personal listening devices [1]. For example, a study by Keppler et al. showed that approximately 30% of 163 young adults exceeded the weekly equivalent noise exposure of 75 dBA in all recreational activities. Furthermore, 86% of their participants reported temporary tinnitus after exposure to recreational noise [2]. The popularity of personal listening devices such as MP3 players has made these devices more sophisticated and easier to wear [3]. Nearly 50% of young people aged 12–35 years in developed countries are exposed to dangerously elevated levels of music through their personal listening devices [1]. Many researchers are also concerned that prolonged exposure to loud music can lead to non-occupational noise-induced hearing loss in the young population [4–6]. Keppler et al. reported that after one hour of listening to pop-rock music through their MP3 players, young participants experienced temporary hearing loss [7]. Nevertheless, with the growing popularity of smartphones whose sound quality is no longer distorted at the maximum volume and an environment in which users are able to download music for free, it is possible to enjoy listening to music anytime and anywhere [8, 9]. Consequently, anyone with a smartphone is vulnerable to hearing loss if they do not take precautions [10]. This phenomenon suggests the need to educate more users of personal listening devices about safe volume levels and good listening habits.

Although many countries have suggested maximum noise exposure levels for personal listening devices to prevent noise-induced hearing loss, they apply only occupational noise-induced hearing loss criteria (i.e., less than 8 h at 85 dBA) to this new risk. The implementation of these criteria has been inconsistent across countries. For example, the U.S. Occupational Safety and Health Administration (OSHA) has limited the daily safe volume level of any sound to below 85 dB for a maximum of eight hours [11]. European recommendations for personal listening devices have been set at 85 dB since 2013 [1]. Canadian federal regulations limit the sound exposure level to less than eight hours at 87 dBA with a 3-dB exchange rate [12]. India’s the Ministry of Environment and Forests proposes 80 dBA for eight hours per day as the permissible exposure level [13]. WHO also recommends the use of personal listening devices for less than one hour per day at no more than 60% of maximum volume [14]. Furthermore, these criteria are far from practical. According to Vogel et al., most of 73 subjects who had listened to loud music through their MP3 player reported that they usually listened to their music at higher volumes in noisy outdoor environments than in quiet situations [15]. They frequently listened to music at the loudest volume in situations with wind, traffic, and/or other people talking [16]. In addition, the users of personal listening devices understood that listening to loud music could cause hearing loss, but they minimized this concern [15]. If they continue listening to music at the loudest volume, their hearing problems or damage will be much worse later and may even eventually result in premature aged-related hearing loss [14]. Several years ago, smartphone manufacturers provided an indicator of the possibility of hearing loss if their users increased the volume to a certain level. However, they still applied different criteria, even for models made by the same company.

The present study aimed to measure the sound pressure levels of four genres of music at the high-volume steps that smartphones propose as a risk for hearing loss and then compare the levels among different types of smartphone. The results offer detailed information for better understanding of the volume steps on personal listening devices and suggestions for the prevention of noise-induced hearing loss among smartphone users.

## Methods

### Selection of smartphone models

Three major companies (Samsung Electronics, Apple, and LG Electronics) that had produced popular mobile devices in the Republic of Korea were chosen. The two latest models of each bland - the Samsung Galaxy S6 and Galaxy Note 3, the Apple iPhone 5S and iPhone 6, and the LG G2 and G3 - were used in this study.

### Selection of music

Three genres of K-pop music (dance-pop, hip-hop, and pop-ballad) and a Billboard pop chart,^1^ which had assorted genres and slightly different rhythms from K-pop music, were chosen. The five most downloaded songs from www.gaonchart.co.kr between July and October 2016 were chosen for each genre. In terms of popularity, the average number of downloads per song was 79,245 (maximum of 236,326; minimum of 19,427).

### Experiment procedures

Initially, the first risk volume step for the six different smartphones was determined. For Samsung’s Galaxy S6 and Galaxy Note 3, the first risk step was determined by the level having the risk sign proposed by the devices. As a result, the first risk step was ninth from the lowest step for the Galaxy S6 and tenth for the Galaxy Note 3. The maximum volume for both devices was the fifteenth step. For the Apple iPhone 5S and iPhone 6, we removed the sound control function in the environmental settings and then activated the volume step from the minimum (step 1) to the maximum (step 16), the same as when they were first released. The two iPhone models began the first risk volume step at the ninth step, and the maximum level was the sixteenth step. Like the Samsung Galaxy S6 and Galaxy Note 3, the LG G2 and G3 models indicated the ninth step as the lowest for the first risk level and the fifteenth step for the maximum volume level, but LG did not provide any control sign or function for a risk volume level in either the device or the software.

**Fig. 1:**
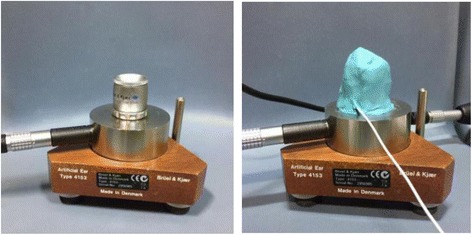
Experi-mental design to measure sound pressure levels using the artificial ear and 2-cm^3^ coupler (left panel) and earplug on the top of the measurement system (right panel)

After deciding the risk volume steps for each device, the sound pressure levels for the music were measured in a sound-treated room. An input plug for earphones (i.e., earbud type; MDR-E9LP, Sony Co., Japan) was connected to the smartphone, and one of two ear tips was connected to a sound analysis system. Since different types of earphones create different levels of sound pressure, even with the same piece of music [10] we used the same earbud for the six smartphones to ensure equal test conditions. The sound analysis system consisted of a 2cm^3^ click-on coupler (Type #4946, Bruel & Kjær, Nærum, Denmark),^2^ an artificial ear (Type #4153, Bruel & Kjær, Nærum, Denmark), a sound level meter (Type #2250, Bruel & Kjær, Nærum, Denmark), and a personal computer [17]. When the music was playing, the sound pressure levels for the earphones could be measured using a sound level meter via the 2cm^3^ coupler on the artificial ear. At the same time, the sound pressure levels were analyzed using computer software (BZ-5503 Measurement Partner Suite, Bruel & Kjær, Nærum, Denmark). Figure 1 depicts the measurement setting followed in the previous publication (See the Fig. 1 of Yu et al.) [17]. The unit of measurement and analysis was the A-weighted decibel (dBA) due to properties of human hearing whereby different frequencies feel like different levels of intensity. The process took approximately 10 h per device.

### Data analysis

Statistical analysis was performed using SPSS software (Ver. 23, IMB Co., Armonk, NY, USA). To evaluate the main effect of smartphones and genres of music and their interaction, a 6 (smartphones) × 4 (music genres) two-way analysis of variance (ANOVA) was conducted. If necessary, Bonferroni corrections were applied with multiple comparisons. The criterion used for statistical significance in the present study was *p*<0.05. In addition, to see how much the sound output levels changed the risk volume steps, graphical analysis was applied as a function of frequency.

## Results

### Comparisons of outputs at the first risk level

ANOVA confirmed a significant main effect for the different kinds of smartphones [F(5, 96) = 47.45, *p* = 0.000]. Among the six phones, the Galaxy S6 showed the lowest output level (mean: 84.1 dBA, SD: 0.43), which differed significantly from the following (in ascending order): iPhone 6 (mean: 88.3 dBA, SD: 0.43), LG G3 (mean: 89.6 dBA, SD: 0.43), Galaxy Note 3 (mean: 89.7 dBA, SD: 0.43), iPhone 5S (mean: 92 dBA, SD: 0.43), and LG G2 (mean: 92.4 dBA, SD: 0.43). Although there was a significant difference between one group iPhone 6, LG G3, and Galaxy Note 3 and another group comprising the iPhone 5S and LG G2, the devices within each group did not differ significantly.

A significant main effect was found for music genre [F(3, 96) = 24.29, *p* = 0.000]. The level of dance-pop (mean: 91.1 dBA, SD: 0.35) was significantly higher than that for the pop-ballad (mean: 89 dBA, SD: 0.35), which in turn was significantly higher than the Billboard pop songs (mean: 87.1 dBA, SD: 0.35). Hip-hop music had the second highest level (mean: 90.2 dBA, SD: 0.35), but this level did not differ significantly from the level for dance-pop. However, it was still higher than the mean for the pop-ballad and Billboard pop songs. A significant interaction was not seen among the types of smartphone and the different music genres [F(15,96) = 1.43, *p* = 0.151].

**Fig. 2:**
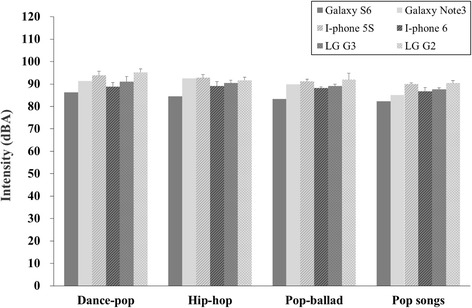
A mean comparison of intensity for six different smartphones for four music genres when the lowest risk volume level (or the first risk volume level) is set. Error bars stand for standard deviation

Figure 2 shows the bar graphs for the average intensity of four genres on six smartphones. The Galaxy S6 had the lowest output level of all music genres (86.3, 84.5, 83.3, 82.4 dBA for dance-pop, hip-hop, pop-ballad, and Billboard pop songs, respectively). The LG G2 showed the highest output level for dance-pop (95.2 dBA), followed by92 dBA for pop-ballad and 90.5 dBA for the Billboard pop songs. Hip-hop music had a level of 92.8 dBA for the iPhone 5S.

### Comparison of sound outputs for consecutively higher levels of risk volume

As the volume step increased, so did the output levels. Table 1 shows the average output levels of 20 songs per device, regardless of music genre. The first risk levels for the six smartphones were, in ascending order, 84.1 dBA for the Galaxy S6, 88.3 dBA for the iPhone 6, 90 dBA for the LG G3, 90 dBA for the Galaxy Note 3, 92 dBA for the iPhone 5S, and 92.4 dBA for the LG G2. However, there was a wider range of maximum output levels. The Galaxy S6 had the lowest at 105.7 dBA, and the iPhone 6 had the highest, at 113.1 dBA. When the WHO recommendation of keeping the volume at 60% of the personal listening device’s maximum volume step is followed, the results of the present study are 63 to 67 dBA.

**Table 1:** Sound outputs at and above the risk volume steps for six different smartphones playing music (a mean of 20 songs).

	RL	RL + 1	RL + 2	RL + 3	RL + 4	RL + 5	RL + 6	RL + 7
Galaxy S6	84.11	87.11	90.11	94.09	97.99	101.89	***105.72***	–
Galaxy Note 3	89.68	93.13	95.47	99.52	103.26	***107.04***	–	–
I-phone 5S	91.97	96.02	96.69	100.06	104.18	107.17	110.08	***112.44***
I-phone 6	88.25	92.05	98.32	101.44	104.49	107.76	110.21	***113.11***
LG G2	92.35	94.90	97.91	100.73	103.34	106.45	***109.42***	–
LG G3	89.60	92.62	95.60	98.43	101.16	104.27	***107.25***	–

Unit: dBA

Risk level (RL) refers to the level of the first risk step that each device suggests. The RL plus number displays the consequent higher volume steps of the first step. Bold and italic style indicates the maximum level for that device

**Fig. 3:**
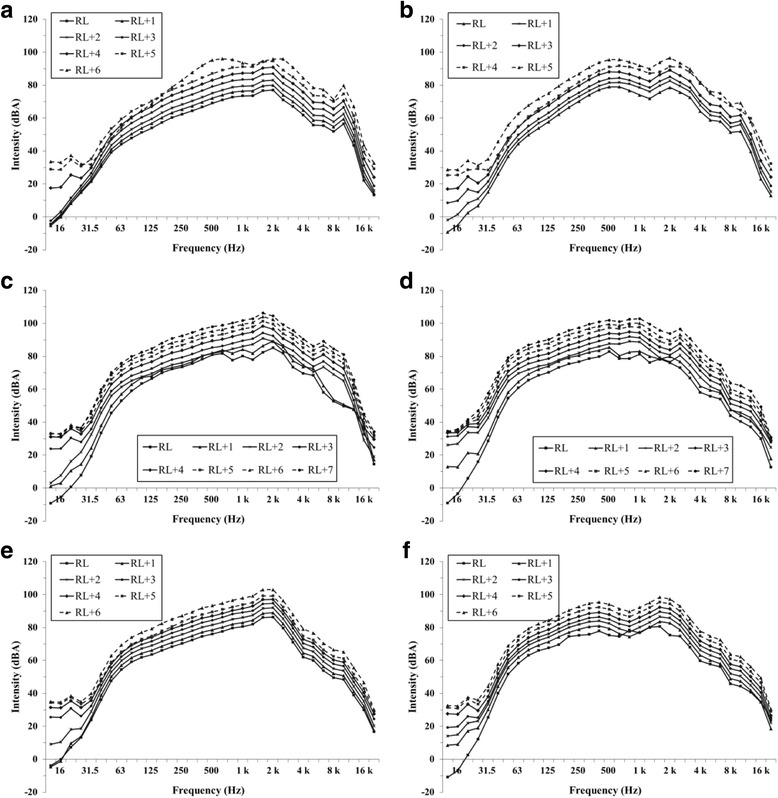
A mean comparison of sound output levels for 20 songs measured at various risk volume steps. Each panel indicates the six smartphones that displayed the first risk volume level (RL) and the levels of their consequent volume steps (RL + 1, RL + 2, ..., RL + 6) displayed as a function of frequency: (**a**) Galaxy S6, (**b**) Galaxy Note 3, (**c**) I-phone 5S, (**d**) I-phone 6, (**e**) LG G2, (**f**) LG G3

In Fig. 3, which indicates the intensity as a function of frequency, the output levels were gradually with the volume steps. A frequency range between 63 and 8000 Hz had higher output levels than either the lower or the higher frequencies. The highest level was 2000 Hz, although Panels B and F in Fig. 3 show double peaks at 500 and 2000 Hz.

## Discussion

This present study measured the output levels of four music genres at the volume steps from the latest models of smartphone proposed as creating a potential risk for hearing loss. The study confirmed a significant 8-dB difference in the sound pressure levels for six kinds of smartphone at the first risk volume level, indicating the need for an international standard for the safe use of personal listening devices. Based on results of this study, the lowest output levels of 84.1–92.4 dBA were enough to produce a temporal threshold shift in hearing level when the users listened to music for a long time, such as eight hours based on current criteria. Since these levels were slightly higher than those of the previous study [16], which were slightly lower than the noise levels from occupational and industrial settings, we should pay more attention to the potential for noise-induced hearing loss [18, 19]. As mentioned in the introduction, the use of occupational criteria to conduct a risk assessment of the use of personal listening devices is not appropriate. That is, the employees who have fixed noise levels and exposure times but have regular break times show a different pattern of loud noise exposure from users of personal listening devices who have large individual differences in terms of levels, duration, and places to listen [16]. As we expected, dance-pop showed the highest output level (91.1 dBA) among music genres in all smartphones, a finding that is supported in previous studies [4, 17] in that there was a significant difference in the output levels for the different music genres. However, various types of earphones should be included and measured in a future study to confirm the current results [10, 20].

The popularity of smartphones, with 383 million devices sold globally in the third quarter of 2017 [21], has resulted in more people than ever using their smartphones as personal listening devices. In practice, Samsung Electronics’ recommended risk volume level is neither enforced not enforceable to its users. Apple Inc., on its official website, also suggests several vague guidelines for the prevention of hearing loss. For example, it states, “if you experience tinnitus, decrease the volume,” and “limit the time when you are listening to music at a high level.” Although Apple Inc. offers software that allows users to limit the maximum volume on the device, most people are not familiar with the software or its function. Only 29.4% of respondents knew about the software, and none had ever used it [15, 16].

At the same time, WHO has campaigned for a safe listening level on personal listening devices, recommending that the volume be set at no more than 60% of the maximum volume [14]. Additionally, WHO has suggested limiting the daily use time; it is very important to keep the volume down and limit the use of personal listening devices to less than one hour a day. When measured at the maximum volume step, our results showed approximately 105–113 dBA, similar to a previous study that showed 91–121 dBA [22]. Thus, 60% of the maximum volume may be estimated as being 63–67 dBA, but users do not always listen at these recommended levels in daily use. According to a recent study by Hoover and Krishnamurti [18], 428 college students who frequently used MP3 players showed unexpected results. Their results, when summarized, indicated that 36.6% of all respondents reported sometimes listening to MP3 players at full volume, and 25.2% listened to music at levels greater than 75% of the maximum volume, which might be a risk factor for noise-induced hearing loss. This result is supported by earlier studies [16, 23]. Thus, we need more practical public education and device modifications to reduce these risk output levels. For example, detailed information generated with a scientific approach while calculating the volume levels of current smartphones and exposure duration and then applying the 3- or 5-dB exchange rate should be considered. Based on our results, which showed different output levels even at the first risk volume, the manufacturers should accurately calculate safe volume levels of MP3 players and smartphones and provide numerical values to the users. If this is done, users of personal listening devices will have a better understanding of daily accumulated noise and the severity of the associated risk to their hearing.

Again, noise-induced hearing loss is the most common form of acquired hearing impairment. However, this kind of hearing loss might be preventable [24, 25]. Many professionals working in the hearing sciences are concerned that noise-damaged ears due to inappropriate listening levels and habits during young adulthood are vulnerable to age-noise interaction and more severe age-related hearing loss. The adverse effects of noise-induced hearing loss accelerate age-related hearing loss for any person who has already experienced noise damage to their ears, as proved by both an animal study [26] and a retrospective clinical study [27]. Therefore, users should be interested in having information on how to limit their exposure to such noise. Another solution would be to encourage users to wear noise-cancelling earphones/headphones that reduce background noise so that music can be heard clearly at lower volumes [14]. Otherwise, users should take frequent breaks from listening to reduce their exposure to noise [1, 14].

Although some limitations to the present study warrant further research in terms of the difference in user gender and type of earphones [20, 28, 29], the results have clear implications for calculating safe levels of smartphone volume and future standardized tools and protocols of daily listening time at those levels. We confirmed that the risk volume steps proposed by the latest models of smartphones are high enough to cause noise-induced hearing loss if their users habitually listen to music at those levels [15], and this risk should be better communicated to users through practical public education and device modification that can help deliver positive restrictions of the sound output levels while still allowing users to enjoy listening to music safely.

## Conclusion

Based on an examination of several kinds of smartphones and genres of music for non-occupational noise exposure, this study measured the sound pressure levels that were at and above the risk volume steps. The results showed that the sound pressure levels of either the first volume step or the maximum volume step were not the same across types of smartphone and genres of music, which means that the risk volume sign and its output levels should be unified across devices. Furthermore, the risk volume steps proposed by the latest smartphone models are high enough to cause noise-induced hearing loss if their users habitually listen to music at those levels.

## Abbreviations

ANOVA: Analysis of variance
OSHA: Occupational Safety and Health Administration
WHO: World Health Organization

## Footnotes

1 Billboard is an American entertainment media brand which publishes news, video, opinion, reviews, events, and style. It is also known for its music charts such as the Billboard Hot 100 consisting of the most popular singles and albums in different genres.

2 2cm^3^ coupler is representative of the ear canal volume when using earbud. It is designed for measurements on all types of hearing aids and is optimized for repetitive use in the laboratory and on the production line while fulfilling the requirements of the ANSI S3.7 (2008) and IEC 60318–5 (2006) standards.
